# Segmental resection of myxoid chondrosarcoma: a case report

**DOI:** 10.3389/fonc.2025.1525039

**Published:** 2025-03-21

**Authors:** Qinghao Liu, Xing Wu, Weiwen Zhao, Junjie Peng, Hao Yin

**Affiliations:** ^1^ The Department of Orthopedics, The First Affiliated Hospital of Hunan Normal University Hunan Provincial People’s Hospital, Changsha, China; ^2^ The Department of Orthopedics, Changsha Hospital of Traditional Chinese Medicine, Changsha, China

**Keywords:** chondrosarcoma, prognostic analysis, imaging examination, vertebral body of the spine, differential diagnosis

## Abstract

We report a rare case of extradural spinal chondrosarcoma in a 48-year-old female. The patient was asymptomatic until a traumatic injury led to lumbar pain, revealing a tumor with atypical imaging characteristics in adjacent vertebral segments. MRI confirmed an extradural spinal tumor, which was surgically excised. Histopathological examination identified the tumor as myxoid chondrosarcoma. The patient underwent extensive surgical treatment, with no local recurrence or distant metastasis observed after five years. Her lumbar pain also completely resolved within the first postoperative year. The management of the rare entity of spinal canal chondrosarcoma is discussed.

## 1 Introduction

Chondrosarcoma is a relatively rare but aggressive malignant bone tumor that primarily affects chondrocytes. It predominantly occurs in males and is most commonly found in the pelvis, proximal femur, proximal humerus, and ribs (1). Occurrence in the vertebral column is exceedingly rare (2). The main subtypes of chondrosarcoma include conventional, dedifferentiated, clear cell, mesenchymal, and myxoid variants, with the myxoid type being one of the rarest. The myxoid subtype of chondrosarcoma is indeed one of the rarer variants, distinguished by its pathology, which shows an abundance of myxoid matrix (3). This case report describes a female patient diagnosed with intradural myxoid chondrosarcoma at the thoracolumbar region. The patient was treated solely by surgical resection without adjuvant radiotherapy or chemotherapy. A five-year follow-up showed no signs of tumor recurrence, providing valuable insights into the clinical diagnosis and management of this rare entity.

## 2 Case report

**Figure 1:**
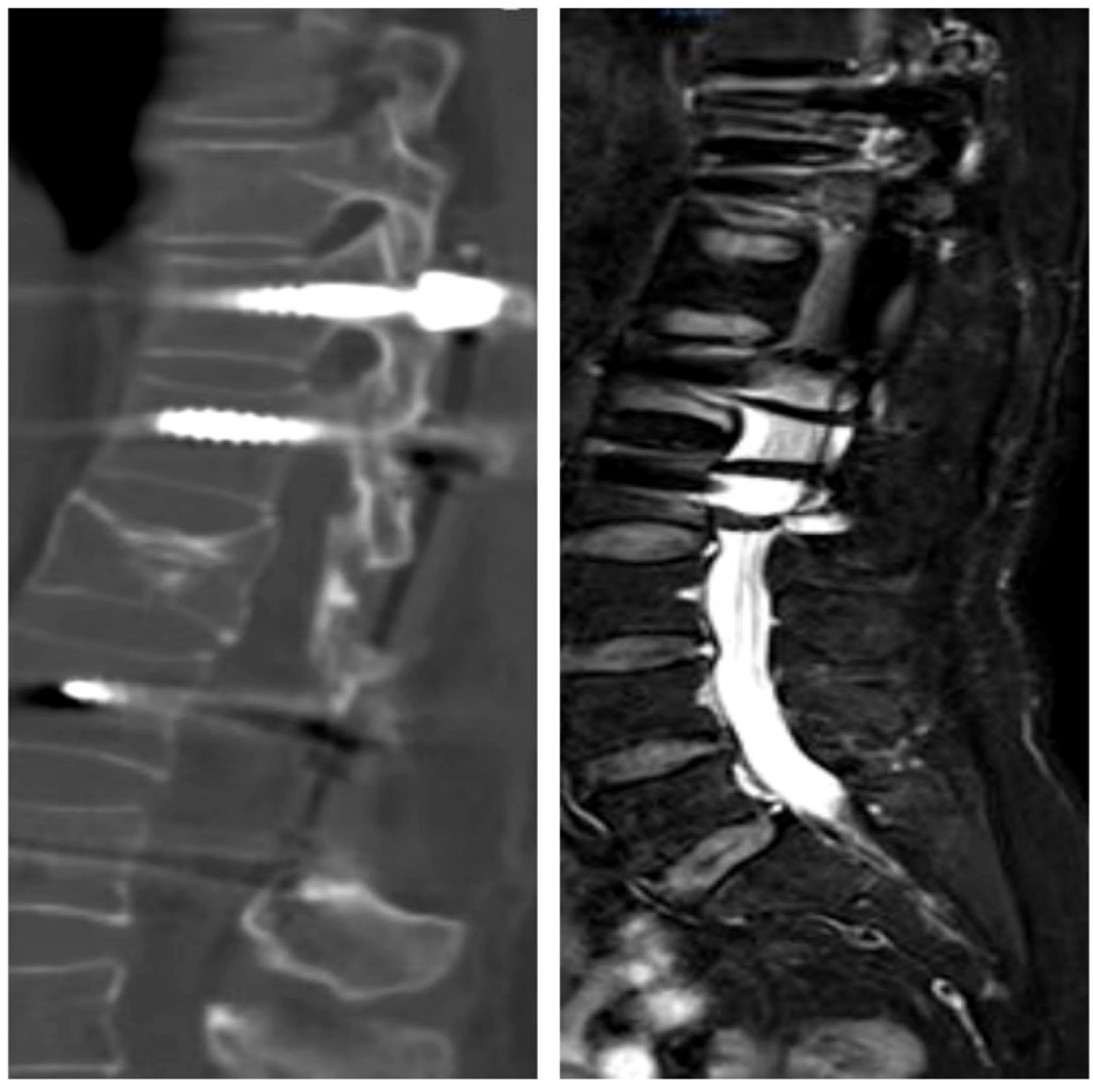
Three-dimensional CT revealed a wedge-shaped deformity of the T12 vertebral body, with no bone fragments within the spinal canal. The L1 vertebral body and its appendages were intact, with no evidence of bone destruction.

**Figure 2:**
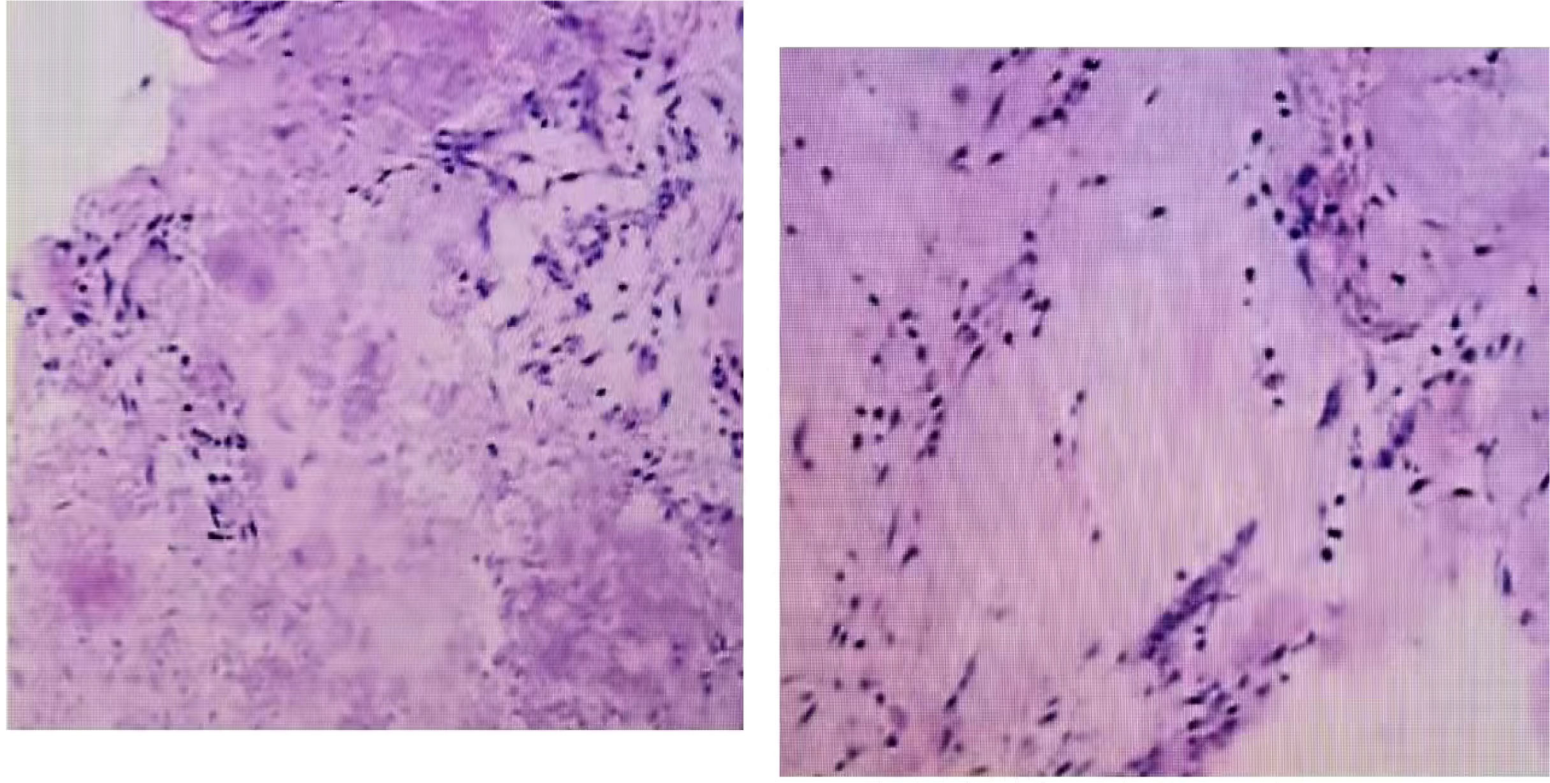
Unenhanced MRI revealed a wedge-shaped deformity of the T12 vertebral body, with patchy areas of long T1, long T2, and T2-weighted high signal intensity, causing mild compression of the corresponding dural sac segment. Contrast-enhanced MRI show.

**Figure 3:**
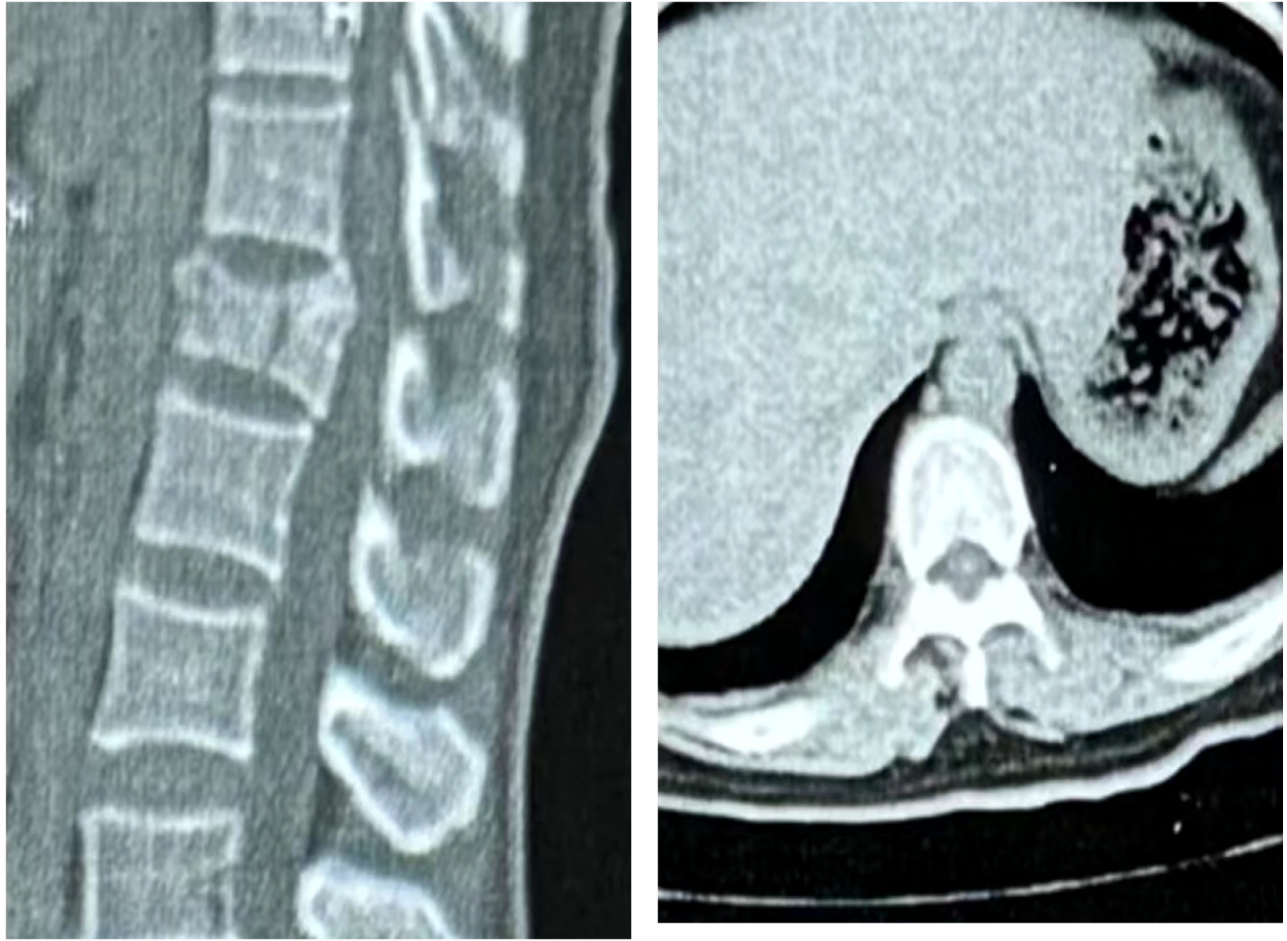
Microscopic examination revealed that hematoxylin-eosin staining showed malignant tumor cells composed of cartilaginous elements, interspersed with round and spindle-shaped cells.

**Figure 4:**
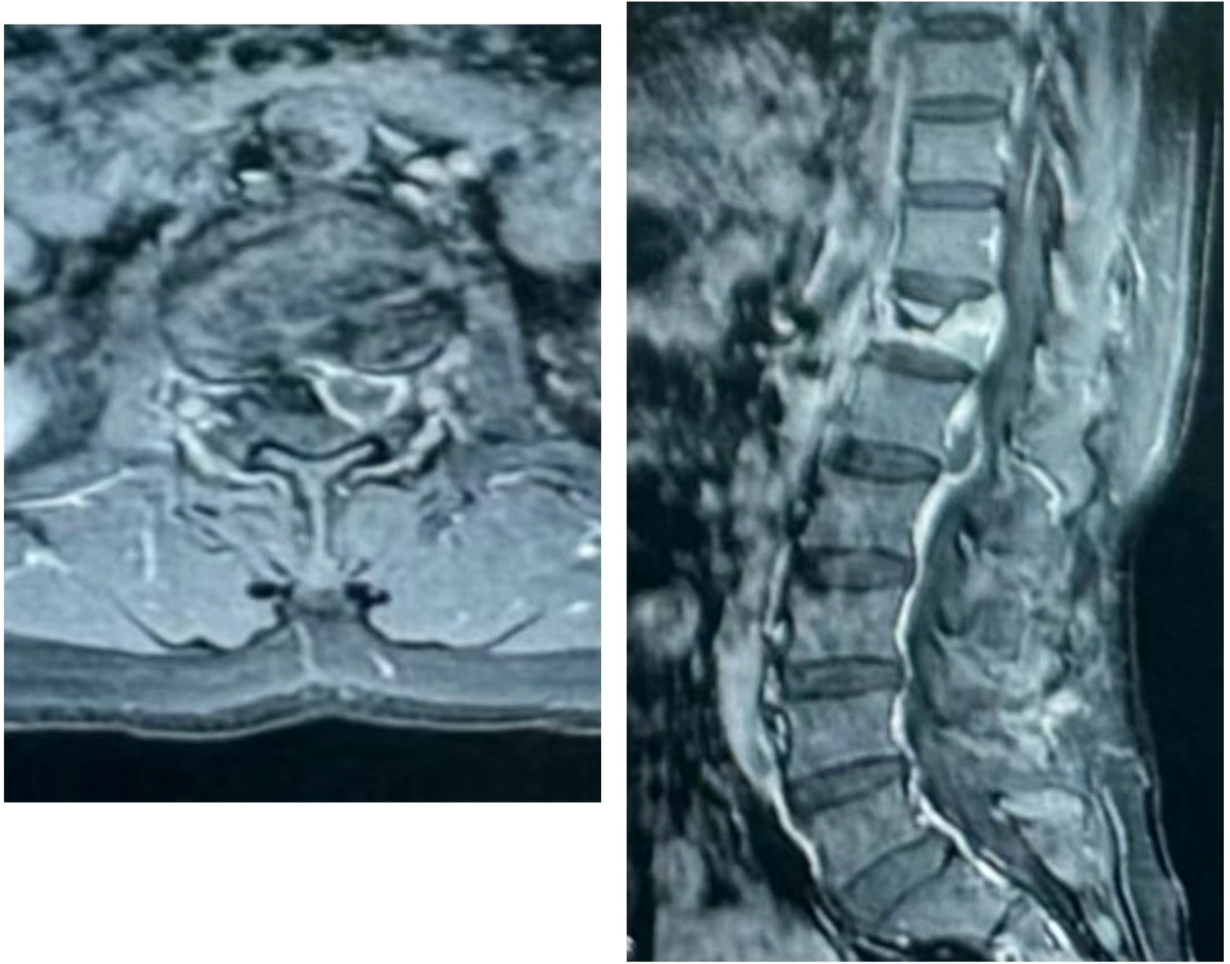
The 5-year postoperative follow-up CT and MRI showed good healing of the T12 vertebral body fracture, with no evidence of bone destruction in the remaining vertebral bodies or their appendages. Additionally, no signs of recurrent soft tissue ma.

The patient, a 48-year-old female, was admitted due to “low back pain and restricted movement for 9 days following a fall.” Physical examination revealed slight muscle tension in the thoracolumbar region, tenderness at the T12 spinous process, and percussive pain in the paraspinal muscles. Bilateral lower extremity muscle strength and tone were normal, and superficial sensation in the trunk and lower limbs was intact. Pathological reflexes, including Babinski sign, Oppenheim sign, Gordon sign, Hoffmann sign, Chaddock sign, and patellar clonus, were all negative bilaterally. A 3D CT scan indicated a wedge-shaped T12 vertebral body with multiple fracture lines, and an intact spinal canal without any abnormal density shadows (**Figure 1**). Enhanced MRI revealed wedge-shaped deformation of the T12 vertebral body, with patchy areas of long T1, long T2, and T2 STIR hyperintense signals, causing mild compression of the corresponding dural sac. A round soft tissue signal shadow, measuring approximately 11 mm × 9 mm with clear boundaries, was observed in the left L1/L2 nerve root path. The lesion exhibited ring-shaped enhancement on contrast imaging, compressing the left nerve root (**Figure 2**). Initial diagnoses were: (1) T12 vertebral compression fracture (AO: A3);(2) Suspected intradural spinal tumor. After ruling out distant metastasis, the patient underwent reduction of the T12 vertebral fracture, pedicle screw fixation, and resection of the intradural mass. Intraoperatively, a mass lesion measuring approximately 1.5 cm × 1.0 cm was identified at the left lower margin of the L1 vertebral body, located extradural, with significant local dural compression. The lesion was solid, without a capsule, well demarcated from surrounding tissues, and of moderate consistency. No obvious tumor tissue was observed intradural. The lesion was completely resected under a microscope and sent for pathological examination. Histopathology revealed atypical myxoid cartilage tissue with focal necrosis, consistent with a diagnosis of well-differentiated chondrosarcoma (**Figure 3**). The patient did not undergo any form of adjuvant chemotherapy or radiotherapy postoperatively. During regular follow-up over the subsequent five years, spinal MRI and CT scans consistently showed no abnormal signal indicative of recurrent lesions in the spinal canal (**Figure 4**).

## 3 Discussion

Myxoid chondrosarcoma typically presents as a low-signal soft tissue mass on imaging, with ring-shaped enhancement following contrast administration. However, these imaging characteristics are nonspecific and may be easily confused with other spinal tumors, particularly schwannomas within the spinal canal. Schwannomas are common neurogenic tumors that arise from Schwann cells of the nerve sheath—a layer of tissue surrounding nerves, with Schwann cells serving as an integral component of this sheath (4, 5). Most schwannomas are benign, with malignancy being exceedingly rare, and they can occur in various locations throughout the body. When schwannomas develop in the spinal canal, pain is often the initial symptom, typically presenting as radiating pain in the shoulder, back, lumbosacral region, or limbs, which worsens during rest. Additional symptoms may include numbness in the extremities, lower limb weakness, gait disturbances, and sensory deficits below the lesion, where strong stimuli may be perceived only as mild sensations. While schwannomas are generally extramedullary, they may extend both inside and outside the spinal canal. On MRI, schwannomas typically exhibit low signal intensity on T1-weighted imaging and mixed low-to-high signal intensity on T2-weighted imaging (6). They are well-circumscribed, rarely exhibit calcification, and often display characteristic features such as a “target sign” or dumbbell-shaped appearance.

In contrast, chondrosarcomas are typically characterized by osteolytic lesions with “ring-and-arc” calcifications of the cartilage matrix on CT imaging. On MRI, these tumors demonstrate high T2 signal intensity due to their cartilaginous and myxoid components, along with peripheral and septal enhancement (7). They are frequently accompanied by the enhancement of extraosseous soft tissue components, with tumor margins and internal regions showing prominent calcification. In the present case, the initial imaging findings were atypical, and the absence of radicular symptoms further complicated the diagnosis. The clinical presentation was masked by the patient’s traumatic compression fracture, making differentiation between benign and malignant tumors particularly challenging.

Despite the diagnostic difficulties posed by differentiating between benign and malignant tumors, timely decision-making regarding treatment strategies and surgical approaches is critical. Evidence from the literature indicates that, compared to intralesional resection, en bloc resection has been proven to significantly improve survival rates as well as neurological and pain-related outcomes in the surgical management of spinal tumors (8). Notably, while en bloc resection with tumor-free margins is considered the gold standard for treating spinal chondrosarcoma, offering longer recurrence-free intervals and improved survival, it presents significant technical challenges. Spinal resection often necessitates navigating the proximity of neurovascular and visceral structures, along with the spine’s complex anatomy, making wide-margin resection difficult to achieve. Even with meticulous preoperative planning, attaining tumor-free margins may not always be feasible (9, 10). Consequently, whether en bloc resection should universally be considered the standard treatment for myxoid chondrosarcoma remains debatable. In this case, the tumor’s atypical location and our limited understanding of its biological behavior led to a decision to perform segmental rather than en bloc resection.

Furthermore, some studies indicate that adjuvant radiotherapy may improve local control rates, although the evidence remains inconsistent (11). Chemotherapy is generally not effective in conventional chondrosarcoma but may play a role in specific subtypes, such as dedifferentiated chondrosarcoma (1, 12, 13). For myxoid chondrosarcoma, a low-grade variant of chondrosarcoma, we did not pursue postoperative adjuvant radiotherapy or chemotherapy. Surprisingly, no recurrence or metastasis was observed during the five-year follow-up period. At the five-year mark, the patient reported full mobility without back pain or the sensory and motor deficits present at admission. This outcome suggests that for early-stage spinal tumors such as myxoid chondrosarcoma, which have low malignancy and present challenges in achieving en bloc resection, segmental resection may be a viable treatment option.

However, more case reports on this rare tumor are needed (14), along with comprehensive analyses of different surgical techniques and long-term survival rates. Such studies would enable a more evidence-based evaluation of patient outcomes following various surgical approaches.

In this case, combined 3D CT and enhanced MRI played a crucial role in determining the tumor’s precise location and characteristics, providing essential preoperative information. With advancements in high-resolution imaging technologies, PET-CT is increasingly utilized for differential diagnosis, further enhancing diagnostic accuracy. The uniqueness of this case lies in the extended five-year postoperative follow-up, which is rarely reported in the literature. Most follow-up studies on chondrosarcoma are relatively short, limiting the ability to assess long-term surgical outcomes. Here, the patient underwent no adjuvant therapy and was monitored solely through routine imaging to detect potential tumor recurrence. Consistently, imaging showed no abnormal signals within the spinal canal, indicating a successful surgical outcome.

This case underscores important diagnostic and therapeutic strategies for myxoid chondrosarcoma of the spine and highlights the significance of multidisciplinary collaboration in managing rare and complex conditions.

## 4 Conclusions

Myxoid chondrosarcoma is a rare and aggressive tumor, and accurate diagnosis along with complete surgical resection is crucial for improving patient outcomes. This detailed case report, combined with a five-year follow-up, provides valuable insights for clinical practice. In the future, more multicenter studies and clinical trials are needed to explore novel therapeutic strategies and optimize follow-up protocols, aiming to enhance the overall treatment outcomes for this rare type of tumor.

## Data Availability

The original contributions presented in the study are included in the article/supplementary material. Further inquiries can be directed to the corresponding author.
